# DNA methylation of candidate genes in peripheral blood from patients with type 2 diabetes or the metabolic syndrome

**DOI:** 10.1371/journal.pone.0180955

**Published:** 2017-07-20

**Authors:** Sanne D. van Otterdijk, Alexandra M. Binder, Katarzyna Szarc vel Szic, Julia Schwald, Karin B. Michels

**Affiliations:** 1 Institute for Prevention and Cancer Epidemiology, University Medical Center Freiburg, Freiburg, Germany; 2 German Cancer Consortium (DKTK), Freiburg, Germany; 3 German Cancer Research Center (DKFZ), Heidelberg, Germany; 4 Department of Epidemiology, Harvard School of Public Health, Boston, MA, United States of America; 5 Obstetrics and Gynecology Epidemiology Center, Department of Obstetrics, Gynecology and Reproductive Biology, Brigham and Women's Hospital, Harvard Medical School, Boston, MA, United States of America; University of Oslo, NORWAY

## Abstract

**Introduction:**

The prevalence of type 2 diabetes (T2D) and the metabolic syndrome (MetS) is increasing and several studies suggested an involvement of DNA methylation in the development of these metabolic diseases. This study was designed to investigate if differential DNA methylation in blood can function as a biomarker for T2D and/or MetS.

**Methods:**

Pyrosequencing analyses were performed for the candidate genes *KCNJ11*, *PPARγ*, *PDK4*, *KCNQ1*, *SCD1*, *PDX1*, *FTO* and *PEG3* in peripheral blood leukocytes (PBLs) from 25 patients diagnosed with only T2D, 9 patients diagnosed with T2D and MetS and 11 control subjects without any metabolic disorders.

**Results:**

No significant differences in gene-specific methylation between patients and controls were observed, although a trend towards significance was observed for *PEG3*. Differential methylation was observed between the groups in 4 out of the 42 single CpG loci located in the promoters regions of the genes *FTO*, *KCNJ11*, *PPARγ* and *PDK4*. A trend towards a positive correlation was observed for *PEG3* methylation with HDL cholesterol levels.

**Discussion:**

Altered levels of DNA methylation in PBLs of specific loci might serve as a biomarker for T2D or MetS, although further investigation is required.

## Introduction

The prevalence of metabolic diseases has been rising worldwide as a consequence of the increasing rate of obesity and sedentary lifestyles[1]. Two prominent metabolic diseases are diabetes mellitus type 2 (T2D) and the metabolic syndrome (MetS). While T2D encompasses insulin resistance, MetS encompasses a more complex network of risk factors and symptoms that predisposes to diabetes and cardiovascular diseases. The symptoms of MetS include elevated blood pressure, dyslipidemia, raised fasting glucose and central obesity[1]. The main risk factors for metabolic diseases, namely low physical activity, genetic predisposition and poor diet[2], are also factors which can modify DNA methylation and other epigenetic patterns[3,4].

DNA methylation is a biological process by which a methyl group is added to a DNA nucleotide, without changing the underlying DNA sequence[5]. The state of DNA methylation of CpG loci near gene promoters, CpG shores and gene bodies were found to be associated with transcriptional activity and gene expression[6–9]. DNA methylation is thought to play a role in regulating human metabolism[10] and epigenetic changes have been suggested to participate in the development of T2D[11]. Several studies have observed differential methylation in T2D and MetS patients compared to control subjects in adipose tissues, muscles and pancreatic islets in genes important for human metabolism[12–16]. Overexpression or silencing of some of these differentially methylated genes resulted for example in reduced β cell proliferation or glucose-stimulated insulin secretion[17,18]. However, to make DNA methylation levels useful as biomarker for metabolic disorders it is essential to identify markers reflected in easily accessible tissues such as blood. Bacos *et al* reported a 60% overlap between genes associated with age-related changes in DNA methylation in both leucocytes and pancreatic islets and some of these differentially methylated genes affected insulin secretion[19] and Vanderjagt *et al* reported that differential DNA methylation in blood was observed in the pre-T2D state[20], suggesting a potential of DNA methylation early in the development of T2D.

We designed this study to investigate gene-specific methylation levels in peripheral blood leukocytes (PBLs) of patients diagnosed with T2D or MetS. The aim of this study was to investigate whether differential DNA methylation was present in PBL of patients diagnosed with either T2D or MetS compared to a control group, and whether altered levels of DNA methylation in blood may function as a biomarker for these metabolic conditions. We used a candidate gene approach including 8 genes that have a functional relevance in metabolic processes, such as glucose metabolism, fatty acid storage, fatty acid metabolism, pancreatic development and β cell maturation[21–26]. Five of these genes, *FTO*, *KCNQ1*, *PDK4*, *PDX1* and *PPARγ*, were previously identified to be differentially methylated in adipose tissues, muscles and/or pancreatic islets from diabetic patients [12–16].

## Methods

### Study population

All participants were recruited at the Freiburg University Medical Center Diabetes Clinic and a written informed consent was obtained from all the participants included in this study. The study was approved by the Albert-Ludwigs-Universität Freiburg ethics committee and Institutional Review Board. Measurements of all metabolic traits were performed by trained physicians at the University Medical Center Freiburg Diabetes Clinic, according to their validated guidelines. The measurements were performed after an overnight fast.

All participants were between 45 and 85 years of age. This study included 11 participants without any metabolic condition and 34 metabolic patients, from which 25 participants were diagnosed with only T2D, and 9 participants were diagnosed with both MetS and T2D. All patients diagnosed with MetS in our study were diagnosed with T2D and had central obesity, plus at least one of the following three factors which defines MetS; elevated triglycerides ≥150 mg/dL (1.7 mmol/L) or specific treatment for this lipid abnormality; reduced HDL cholesterol <40 mg/dL (1.03 mmol/L) in males and < 50 mg/dL (1.29 mmol/L) in females or specific treatment for this lipid abnormality; or raised systolic blood pressure ≥130 or diastolic blood pressure ≥85 mmHg or treatment of previously diagnosed hypertension[27]. None of the participants were diagnosed with another condition, such as cancer or autoimmune disease. Further characteristics of the study participants can be found in Table 1.

**Table 1:** Study participant characteristics. Characteristics of the participants in this study are shown as median (range). The participants were divided into three subgroups: control subjects, T2D patients and MetS patients. The Kruskal-Wallis Test was performed to test for differences between the three groups. The Wilcoxon Rank-Sum Test was performed to test for differences between two groups of participants.

		Median Level (range)			Overall Test (p-value)	Pairwise-Test (p-value)			
Characteristic of study participants		Control	T2D	MetS		Control vs T2D&MetS	Control vs T2D	Control vs MetS	MetS vs T2D
**Age (Years)**		71 (47–85)	69 (50–85)	76 (45–81)	0.99	0.93	0.99	0.85	1.00
**Gender**	**Female**	7 (64%)	10 (40%)	5 (56%)	0.50	0.34	0.27	0.79	0.51
	**Male**	4 (36%)	15 (60%)	4 (44%)					
**Fasting blood glucose (mg/dl)**		84 (69–100)	158 (99–351)	97 (87–158)	<0.01*	<0.01*	<0.01*	0.01*	<0.01*
**Diastolic blood pressure (mmHg)**		80 (60–90)	80 (50–100)	80 (70–90)	0.85	0.73	0.87	0.53	0.72
**Systolic blood pressure (mmHg)**		130 (110–150)	140 (100–170)	135 (120–170)	0.30	0.13	0.16	0.20	0.98
**HDL cholesterol (mg/dl)**		59 (26–92)	40 (15–69)	55 (33–91)	0.01*	0.01*	<0.01*	0.54	0.05*
**Triglycerides (mg/dl)**		109 (65–209)	190 (100–637)	164 (116–285)	0.01*	<0.01*	<0.01*	0.03*	0.60
**Waist measurement (cm)**		86 (69–103)	110 (89–152)	92 (82–110)	<0.01*	<0.01*	<0.01*	0.06	0.01*

*p≤0.05

Age distribution, gender distribution and blood pressure were not significantly different between the groups of participants. As expected, statistically significant differences were observed between the participants diagnosed with T2D and/or MetS and the control group for TG levels, HDL cholesterol levels, waist circumcise and blood glucose levels (p≤0.05)(Table 1). TG levels, HDL cholesterol levels, blood glucose levels and waist measurements were correlated with each other, such that an individual with low levels of HDL cholesterol also had high levels of fasting blood glucose, high TG levels, and a high waist circumference. Similar correlations were found between systolic blood pressure and both diastolic blood pressure and blood glucose levels (p<0.05) (S1 Fig).

### DNA preparation and PCR conditions

DNA was extracted from PBLs using the QIAamp DNA Mini Kit (Qiagen, Hilden, Germany) and genomic DNA was treated with bisulphite salt using the EZ DNA Methylation Gold kit (Zymo Research, Irvine, CA, USA), according to manufacturer's recommendation. The samples were amplified in 20μl reactions, containing 10μl of Hot StarTaq Master Mix (Qiagen, Hilden, Germany), 150ng of each primer and ~ 20ng modified DNA.

PCR was performed with one cycle of 95°C for 15 min, 40 cycles of 95°C for 30 sec, 57–63°C for 30 sec and 72°C for 30 sec, followed by one cycle of 72°C for 5 min. For each set of primers one of the primers included a 5’-biotin label to allow subsequent analysis by pyrosequencing (S1 Table).

Primer sequences were either previously published[12,28–30] or designed using the primer design program ‘PSQ assay’ (Biotage), using gene sequences that were obtained from the GenBank entry on NCBI. Primers were designed near the transcriptional start site of the gene and in an area free of SNPs. One pair of primers was designed per gene. CpG coverage varied between 3 and 9 loci per gene within a length of maximum 41 basepairs (S1 Table). Our candidate genes were located on different chromosomes. *KCNJ11* and *KCNQ1* can be found on chromosome 11, with our primers covering chromosomal positions 17,411,485–17,411,507 and 2,721,948–2,721,988, respectively. The *PPARγ* primers covered the area that can be found on chromosome 3:12,288,073–12,288,089, *PDK4* on chromosome 7:95,584,377–95,584,395, *SCD1* on chromosome 10:100,347,893–100,347,934, *PDX1* on chromosome 13:27,920,513–27,920,543, *FTO* on chromosome 16:53,704,013–53,704,036 and *PEG3* on chromosome 19:56,810,083–56,810,100. For an overview of the primer information and specific CpG positions, see S1 Table.

### DNA methylation analysis

Pyrosequencing was performed on a PyroMark Q24 MD pyrosequencer (Qiagen, Hilden, Germany), according to manufacturer’s recommendation. Pyrosequencing analyses were performed in duplicate. If the duplicates of the individual samples showed a difference < 2% of methylation, the average methylation of the two measurements was used for further analyses. When the difference was >2% a third measurement was performed.

Assay validation was carried out on samples of known methylation status, which were created from whole genome amplified DNA, representing hypomethylation, and DNA treated with CpG methyltransferase *M*.*SssI*, representing hypermethylation (New England Biolabs, Ipswich, MA, USA). Each PCR plate contained the participants’ DNA samples, as well as three dH_2_O samples as non-template controls and three samples with known methylation status as positive controls. The quality of the pyrosequencing assays were analysed using the internal control of the pyrosequencer and the pyrograms. Reproducibility of the pyrosequencing runs was high for all the assays included in this study.

### Statistical analysis

Gene methylation was calculated by determining the average methylation level across the CpG loci per gene. Methylation data were not normally distributed and therefore the nonparametric Kruskal-Wallis test was used to identify significant differences between the three subgroups and the Wilcoxon Rank-Sum Test to perform pairwise comparisons between two groups. These analyses were performed on both the average gene methylation data and the methylation data of the individual CpG loci. A Spearman correlation was performed to identify correlations between measurements. All analyses were conducted using R version 3.2.2 software and visualized using the ggplot2 package[31].

The methylation analyses were performed without prior knowledge of the participant characteristics.

## Results

### Differential DNA methylation between patient groups

**Fig 1:**
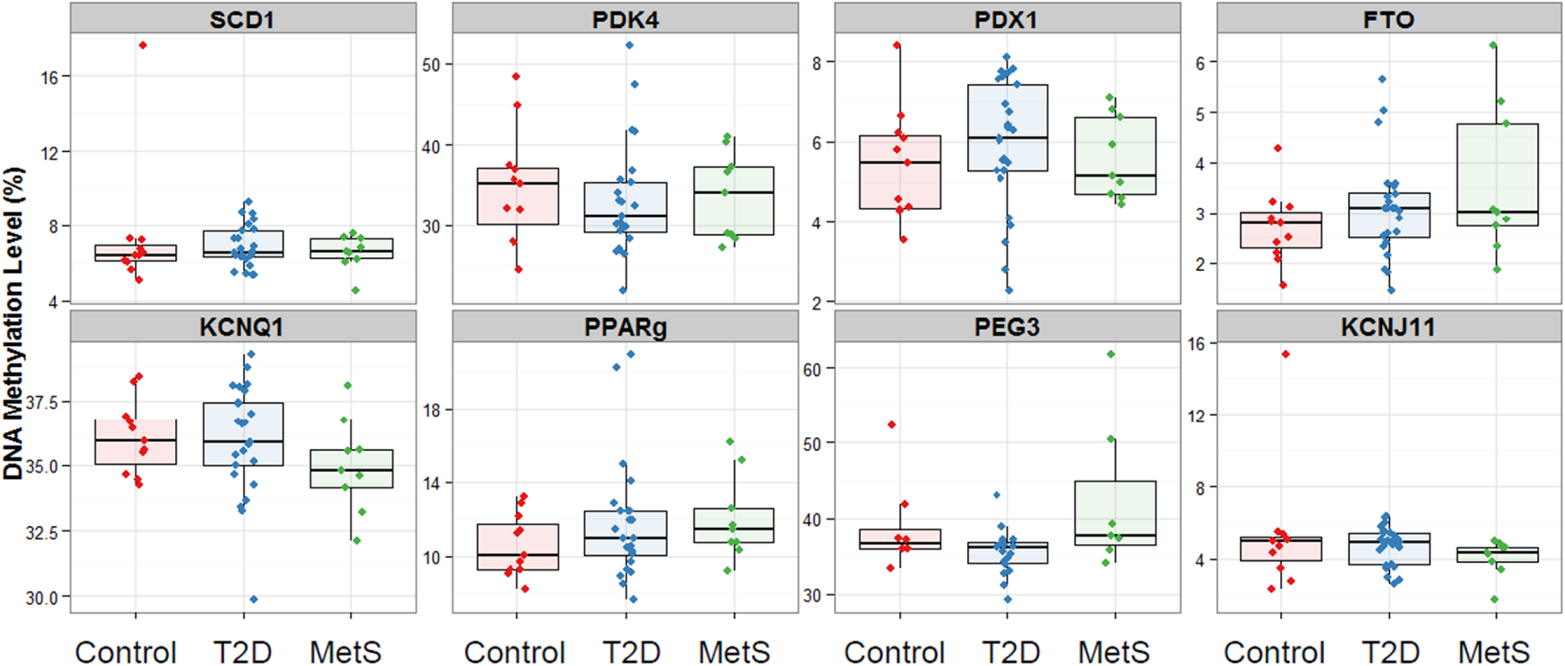
Differences in candidate gene methylation between controls, T2D and MetS participants. Overall gene specific DNA methylation levels are shown per group of participants.

Differences in DNA methylation levels between the three subgroups were small and did not reach statistical significance for any of the genes, although a trend towards significance was observed for the *PEG3* gene (p = 0.07) (Fig 1 and Table 2). A similar trend toward significance was observed for this gene between the controls and all metabolic patients (i.e. the MetS and T2D patients) (p = 0.07). Further analyses of *PEG3* methylation revealed that significantly higher levels of methylation of 1.5 percentage points were observed in the MetS patients compared to the T2D patients (p = 0.03). These statistical differences in *PEG3* methylation were mainly driven by three outliers with methylation levels greater than 50%; two MetS patients and one control subject. Excluding these outliers resulted in a loss of statistical significance, although a trend towards significance was still observed (p = 0.051). Interestingly, the two MetS patients with the highest levels of *PEG3* methylation also had high levels of *PDX1* and *FTO* methylation.

**Table 2:** Differences in candidate gene methylation between controls, T2D and MetS participants. Gene specific DNA methylation levels are shown as median (range). The Kruskal-Wallis Test was performed to test for differences between the three groups. The Wilcoxon Rank-Sum Test was performed to test for differences between two groups of participants.

	Median Methylation Level (range)(%)			Overall Test (p-value)	Pairwise-Test (p-value)			
Gene	Control	T2D	MetS		Control vs T2D&MetS	Control vs T2D	Control vs MetS	MetS vs T2D
*FTO*	2.8 (1.6–4. 39)	3.0 (1.4–5.6)	3.0 (1.9–6.3)	0.40	0.32	0.24	0.26	0.79
*KCNJ11*	5.0 (2.2–15.7)	4.7 (2.4–6.3)	4.3 (1.9–5.0)	0.21	0.87	0.77	0.16	0.10
*KCNQ1*	36.0 (34.2–38.4)	35.9 (29.9–39.3)	34.8 (32.1–38.1)	0.28	0.43	0.97	0.18	0.15
*PDK4*	35.3 (24.5–48.3)	31.2 (21.9–52.2)	34.0 (27.2–40.9)	0.68	0.64	0.39	0.94	0.68
*PDX1*	5.5 (3.5–8.4)	6.1 (2.3–8.1)	5.2 (4.4–7.1)	0.47	0.20	0.36	0.46	0.36
*PEG3*	36.6 (33.5–52.3)	36.1 (29.2–43.1)	37.7 (34.2–61.8)	0.07	0.07	0.18	0.54	0.03*
*PPAR****γ***	10.1 (8.2–13.2)	11.0 (7.6–21.0)	11.5 (9.2–16.2)	0.41	0.12	0.41	0.20	0.49
*SCD1*	6.4 (5.1–17.6)	6.67 (5.3–9.3)	6.6 (4.5–7.6)	0.66	0.30	0.42	0.57	0.69

*p≤0.05

Further investigation into individual CpG loci revealed no statistical differences in DNA methylation levels between the T2D and MetS patients and the control participants. However, after dividing the patients into the MetS or T2D subgroups, some loci were observed to be differentially methylated. These specific CpG loci were found in the *FTO*, *KCNJ11* and *PPARγ* genes. One CpG locus out of the 7 loci in the *FTO* gene indicated differential methylation between the MetS patients and the controls (p = 0.01), with median methylation levels of 2.7% and 2.1% respectively, while another CpG locus in the *FTO* gene was tending toward significance between the T2D patients and the controls (p = 0.07). 1 CpG locus out of the 4 loci in the *PPARγ* gene was significantly higher methylated in the T2D patients compared to the controls, with an average increase of 3.5 percentage points in the T2D patient group (p = 0.05). One out of the 3 loci in the *KCNJ11* gene was differentially methylated between the T2D and MetS patients. For this CpG locus, the MetS patient group had DNA methylation levels that were on average 1.4 percentage points higher than the methylation levels observed for the T2D patient group (p<0.01). In comparison, the control group had median methylation levels that were on average 1.3 percentage points lower than the MetS group and was tending towards significance (p = 0.08)(Table 3). One CpG locus out of the 4 loci in the *PDK4* gene was significantly lower methylated in the T2D and MetS patient groups than in the control group (p = 0.048). The T2D and MetS patient groups had average methylation levels that were 5.7 percentage points lower than the average methylation levels observed in the control group. A trend towards significance was observed for this CpG locus between the control group and the patient group that was diagnosed with only T2D (p = 0.05), but not with the group that was diagnosed with MetS (p = 0.15)(Table 3). A trend towards a significant difference was observed for another Cpg locus in this gene between the control group and the T2D and MetS patient groups (p = 0.06). This trend towards significance was also observed when comparing the control group to the MetS group (p = 0.05), but not when comparing the control group to the group that was diagnosed with only T2D (Table 3).

**Table 3:** DNA methylation levels of individual CpG loci located in the *KCNJ11*, *PPARγ*, *FTO* and *PDK4* genes. DNA methylation levels of specific CpG loci are shown as median (range). The Kruskal-Wallis Test was performed to test for differences between the three groups. The Wilcoxon Rank-Sum Test was performed to test for differences between two groups of participants. DNA methylation levels of individual CpG loci, located in the genes *KCNJ11*, *PPARγ*, *FTO* and *PDK4* were significantly different between groups of participants (p**≤**0.05). None of the other CpG loci included in this study were significantly different between our groups of participants (S2 Table).

	Median Methylation Level (range)(%)			Overall Test (p-value)	Pairwise-Test (p-value)			
CpG loci	Control	T2D	MetS		Control vs T2D&MetS	Control vs MetS	Control vs T2D	MetS vs T2D
***KCNJ11* CpG2** (Chr11:17,411,502)	6.9 (6.0–9. 9)	6.8 (5.7–8.6)	8.2 (6.2–10.2)	0.17	0.34	0.08	0.27	<0.01*
***PPARγ* CpG1** (Chr3:12,288,073)	7.0 (5.6–12.5)	10.5 (6.5–15.0)	9.2 (5.0–21.3)	0.20	0.13	0.18	0.05*	0.66
***FTO* CpG5** (Chr16: 53,704,031)	2.3 (1.2–5.4)	3.1 (1.0–7.7)	2.8 (1.6–6.3)	0.20	0.19	0.13	0.07	0.74
***FTO* CpG6** (Chr16: 53,704,034)	2.1 (0.9–2.8)	2.3 (0.9–7.2)	2.7 (1.2–7.8)	0.19	0.32	0.01*	0.11	0.21
***PDK4 CpG1*** (Chr7: 95,584,377)	56.8 (38.1–80.2)	50.7 (34.8–81.6)	53.2 (45.2–64.9)	0.37	0.05*	0.15	0.05	0.94
***PDK4* CpG4** (Chr7:95,584,395)	27.5 (20.4–37.4)	23.4 (18.2–63.4)	25.9 (20.4–32.8)	0.64	0.06	0.05	0.12	0.27

*p≤0.05

None of the other CpG loci included in our analyses reached statistical significant differences in DNA methylation levels between our groups of participants (S2 Table).

### Correlations between DNA methylation and characteristics of study participants

**Fig 2:**
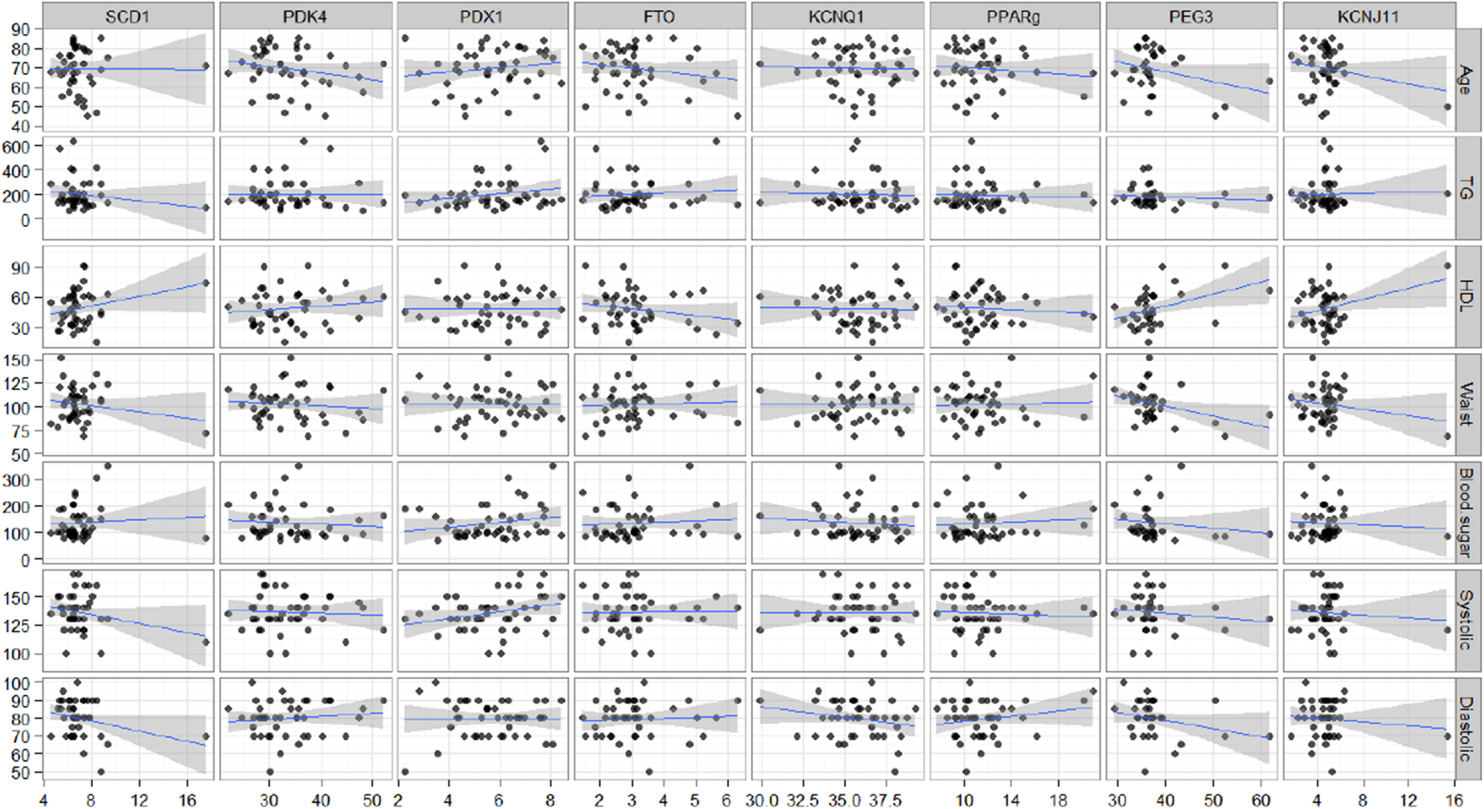
Correlations between DNA methylation and characteristics of study participants. Gene specific DNA methylation levels are shown relative to patient characteristics.

To study a possible correlation between DNA methylation levels and characteristics of study participants, a Spearman’s correlation was performed (Fig 2). These analyses revealed statistically significant correlations between HDL cholesterol, waist measurements and fasting blood glucose levels and *PEG3* methylation (Spearman’s correlation, p<0.05) such that individuals with higher levels of HDL cholesterol, lower levels of fasting blood glucose levels, and lower waist measurements had higher methylation levels of *PEG3*. Further analyses revealed that these correlations were mainly driven by the three outliers with methylation levels greater than 50%. Two of these three outliers were located in the highest quadrantile for HDL cholesterol, and all three were in the lower quadrantile for both waist measurement and fasting glucose levels. Exclusion of these outliers resulted in a loss of statistical significance, although a positive trend remained between *PEG3* methylation and HDL cholesterol (p = 0.05). Further analyses revealed that the correlation between *PEG3* methylation and HDL cholesterol was strongest in the control group (R^2^ = 0.743).

None of the other genes revealed a correlation between methylation levels and either one of the characteristics of the study participants assessed.

## Discussion

In this study we analysed DNA methylation levels of a subset of candidate genes in PBL from patients diagnosed with T2D or MetS and control subjects without a metabolic disorder. All of the genes analysed in this study were previously reported to have a potential importance in the development of metabolic diseases and play critical roles in glucose metabolism, adipogenesis and/or insulin secretion[21–26]. Differential methylation was observed in previous studies for most of these genes between patients diagnosed with either T2D or MetS and control subjects in adipose tissues, muscles and/or pancreatic islets[12–16,28]. Our analyses in PBLs revealed no significant difference in methylation levels between the MetS patients, T2D patients and controls subjects, although a trend towards significance was observed for *PEG3*. A trend towards a positive correlation was also observed for *PEG3* methylation with HDL cholesterol levels. *PEG3* is a mediator of p53, which in turn directly influences various metabolic pathways, enabling cells to respond to metabolic stress. These functions are likely to be important for restraining the development of cancer but may also have a profound effect on the development of metabolic diseases, including diabetes. p53 plays a role in central carbon and lipid metabolism and it downregulates HDL[32,33]. As a mediator of P53, *PEG3* methylation may contribute to the development of metabolic dysfunction by altering the response of cells to metabolic stress. Our study suggests that altered *PEG3* DNA methylation may be present in PBL of patients with a metabolic disorder. Unfortunately our study only included 45 individuals, so additional studies are necessary to explore this relation in greater detail.

Even though none of the genes revealed statistically significant gene specific differences in DNA methylation between the groups of participants, differences were observed in individual CpG loci. For example, methylation of one locus in the *KCNJ11* gene was significantly higher in the MetS group than in the T2D group and was tending towards significance compared to the control group. Genetic defects in the *KCNJ11* gene have been previously associated with an increased risk of diabetic disorders and heart failure, by inducing changes in the heart's response to stress[34,35]. In line with these reports, we could speculate that the elevated levels of DNA methylation in the MetS patient group for this *KCNJ11* CpG locus may mimic the genetic defects in the *KCNJ11* gene. We do not have detailed information about the genetic predispositions of our study population, so this remains speculation and further investigation is required to study this potential in greater detail, although similar mechanisms have been observed in cancer, where hypermethylation in sporadic cancers is frequently observed in the same genes that are mutated in familial cancers [36–39].

DNA methylation levels of one CpG locus in the *PPARγ* gene was significantly increased in the T2D group compared to the control group. Animal knock-out studies have suggested that *PPARγ* contributes to insulin resistance and *PPARγ* agonists are used in the treatment of T2D[40–42]. A study by Davé *et al* reported that correlations between *PPARγ* DNA methylation and *PPARγ* expression were observed for several CpG loci located in the *PPARγ* gene, even for loci located in the gene body[43]. As a result, reduced gene activity of the *PPARγ* gene may be another possible pathway in which *PPARγ* affects the development of T2D. Even though all of our MetS patients were diagnosed with T2D, a similar trend was not observed between the MetS patients and the control subjects.

One CpG locus located in the *PDK4* gene was significantly differentially methylated between our T2D and MetS patients and the control group, while another CpG locus was tending towards a significant difference. DNA methylation levels in the T2D and MetS patient groups were lower than the DNA methylation levels observed in the control group for both of these loci. *PDK4* is reported to contribute to the regulation of glucose metabolism and mitochondrial function and lower levels of *PDK4* DNA methylation, in combination with increased levels of *PDK4* expression, were previously observed in skeleton muscle of diabetic patients compared to a control group[15]. Our results suggest that similar alterations of DNA methylation patterns may also be present in PBL.

Another CpG locus in our study with significantly increased levels of DNA methylation in the MetS patients compared to the control subjects was located in the *FTO* gene, while another locus in this gene was tending towards a significant difference between the control group and the T2D patients. A previous study performed by Toperoff *et al* suggested that *FTO* was differentially methylated in PBLs from diabetic patients compared to non-diabetic patients in an epigenome-wide association study[44]. Another study by the same group suggested that a methylation difference between diabetic patients and controls for one CpG locus located in a region within an intron of the *FTO* gene, was apparent in young subjects, but no longer existed at older ages[45]. Even though our study investigated a different *FTO* region than the study by Toperoff *et al*, and our study population was remarkably older, we did observe differential DNA methylation in a CpG locus located in the *FTO* gene, suggesting a potential of *FTO* gene methylation in PBL as a biomarker of T2D or MetS.

None of the other CpG loci included in our study revealed differential levels of DNA methylation in PBL between our groups of participants. This is in line with a previously published study by Arner *et al*, who performed an epigenome-wide association study including peripheral blood mononuclear cells from 80 obese female participants with and without insulin resistance[46]. Arner *et al* did not observe significant differentially methylated loci between those individuals identified with and without insulin resistance, although some loci were nominally significant[46]. From those nominally differentially methylated loci, one locus was located in the gene body region of *KCNQ1*, while none of the other loci were located in close proximity of our investigated gene regions.

Most of the candidate genes included in our study were previously reported to show differential levels of DNA methylation in other tissues. For example, increased levels of *PDX1* DNA methylation, in combination with decreased *PDX1* expression levels, were observed in pancreatic islets from patients diagnosed with T2D compared to a control group[12], while we did not observed differences in DNA methylation levels for this gene between our T2D diabetes patients and controls in PBL. This suggests that altered levels of DNA methylation for these genes may be tissue type specific.

There are a few limitations in our study which could also have affected our findings. First of all, we have only measured between 3 and 9 CpG loci per gene and it is possible that other CpG loci located in our candidate genes are differently methylated between our groups of participants. For example, in a genome-wide association study, differentially methylated loci were observed in PBLs from pooled T2D patients versus controls in a genome-wide association study for 3 loci located in the intronic regions of the *KCNQ1*[44], while none of the CpG loci included for this gene in our study reached statistical significance. Secondly, with our study population having a mean age of 70 years, it is well possible that larger differences in methylation levels between the different groups would have been observed in a younger population, where age-related decline of metabolic functions might not yet be as substantial. The correlation between age and DNA methylation is well established[47] and Toperoff *et al* reported a loss of significant differences in DNA methylation levels of the *FTO* gene between T2D patients and controls with an increasing age[45]. As a result, it is possible that differential DNA methylation between the T2D and MetS patients and controls of our candidate genes would be more apparent in a younger population. Thirdly, our study population included only 45 participants and may thus be too small to detect differential DNA methylation and strong associations with characteristics of study participants. We have also only investigated a smaller number of genes and CpG loci in this study and the difference in DNA methylation levels between the groups of participants was small. As a result, it is possible that our findings were due to random sampling errors and are not biologically reproducible.

Further investigation into differential methylation between metabolic patients and healthy controls remains necessary and epigenome-wide analyses could reveal other genes with altered levels of methylation in T2D or MetS in PBLs. Several other genes, including *CIDEP*, *FABP3*, *FFAR*, *CCL2* and *PRKCZ*, have previously been identified to be differentially methylated in PBLs from diabetic patients[48–51] and could thus be of potential in the identification of metabolic diseases. Associations between altered levels of DNA methylation and gene expression were observed in T2D patients in several tissues, including PBL[13,16,51,52], which further supports the potential role of DNA methylation in metabolic diseases.

Our current observations do not allow determining the timing of the observed differential methylation. As a result, it remains unknown whether differential methylation is present before the development of the disease, and thus potentially plays a role in the development, or if it is merely a consequence of the disease. One study observed differential DNA methylation in blood in the pre-T2D state[20], suggesting that DNA methylation may be precoding T2D, and thus be present before the development of the disease. This suggests that differential methylation in PBLs might be a hallmark of T2D and MetS and may be used as a tool to identify these metabolic conditions. Differences in methylation levels between T2D and MetS patients were present in one *KCNJ11* locus included in our study, suggesting that in patients with increased insulin resistance, or who are already diagnosed with T2D, DNA methylation might be used as a biomarker for other metabolic conditions, such as MetS. Although this associations was only found in a single loci and further study is necessary to determine to strength of this association.

In conclusion, our study reports a trend towards significance between *PEG3* DNA methylation levels in PBLs in the MetS and the T2D patients versus the controls and 4 out of the 42 CpG loci included in this study were differentially methylated between groups of participants. As a result, altered levels of DNA methylation in PBLs of these loci might serve as a biomarker for T2D or MetS, although additional research is required to further strengthen these observations.

## Supporting information

S1 Fig Correlations between study participant characteristics. The lower triangle contains pair-wise characteristic of study participant scatterplots.
The upper triangle shows the spearman correlation between characteristic of study participants. The colour corresponds to the strength of the correlation, with the dark red colour indicating the strongest correlation.
*p<0.05, **p<0.001.
(TIF)

S1 Table Primer and gene information. Primer sequences and CpG locations for the loci included in this study.
(DOCX)

S2 Table DNA methylation levels of individual CpG loci. DNA methylation levels of specific CpG loci are shown as median (range). The Kruskal-Wallis Test was performed to test for differences between the three groups. The Wilcoxon Rank-Sum Test was performed to test for differences between two groups of participants.
*p≤0.05.
(DOCX)
