# Supplementary figures and images for: DNA methylation of candidate genes in peripheral blood from patients with type 2 diabetes or the metabolic syndrome
Source: PLoS One. 2017 Jul 20;12(7):e0180955. doi: 10.1371/journal.pone.0180955 (PMC5519053; doi:10.1371/journal.pone.0180955)

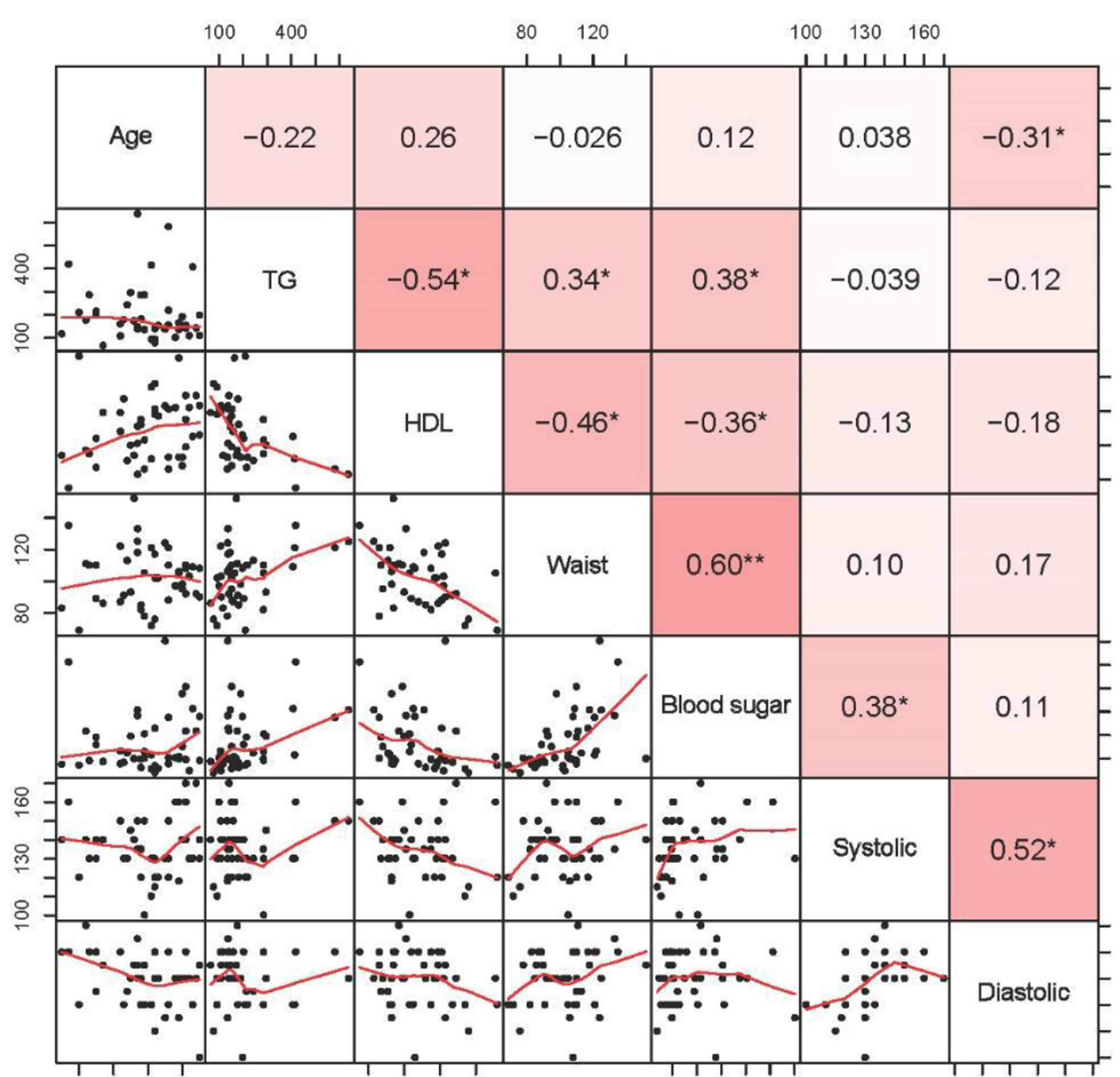

Supplement: S1 Fig — The lower triangle contains pair-wise characteristic of study participant scatterplots. The upper triangle shows the spearman correlation between characteristic of study participants. The colour corresponds to the strength of the correlation, with the dark red colour indicating the strongest correlation. *p<0.05, **p<0.001. (TIF) [file pone.0180955.s001.tif]
