# Supplementary material for: DNA methylation of candidate genes in peripheral blood from patients with type 2 diabetes or the metabolic syndrome
Source: PLoS One. 2017 Jul 20;12(7):e0180955. doi: 10.1371/journal.pone.0180955 (PMC5519053; doi:10.1371/journal.pone.0180955)
Supplement: S1 Table — Primer sequences and CpG locations for the loci included in this study. (DOCX) [file pone.0180955.s002.docx]

S1 Table. Primer and gene information

| Gene | Primers | CpG coverage | Position CpG loci | Distance from TSS | Reference |
| --- | --- | --- | --- | --- | --- |
| *KCNJ11* | Predesigned primers Qiagen (PM00156121) | 3 | Chr11:17,411,485; Chr11:17,411,490; Chr11:17,411,507 | -409  -404  -387 |  |
| *PPARγ* | F: 5’-TTGGAAAGAATATTTTGGGAAGA-3’  R: 5’-Biotin-ACCCAAAAAAAATCCCATTTC-3’  S: 5’-GAATATTTTGGGAAGA-3’ | 4 | Chr3:12,288,073; Chr3:12,288,082;  Chr3:12,288,086;  Chr3:12,288,089 | +128  +137  +141  +144 | [29] |
| *PDK4* | F: 5’-GTTTGGTGTTTYGAGAGGTGGAGTAT -3  R: 5’Biot-CCCTCTCACCAAAATCCAATAACTACTTC-3  S: 5’-AGAGGTGGAGTATTTTT-3 | 4 | Chr7: 95,584,377;  Chr7:95,584,379;  Chr7:95,584,389; Chr7:95,584,395 | +291  +293  +303  +309 | [30] |
| KCNQ1 | F: 5’-ATTTTAGGGGGTGAGTGGTA-3  R: 5’-BiotinACTTTTATAACCCAAACTTTTAT  CCC-3  S: 5’-AGGTTATTTATTTGGTAAAGG-3 | 9 | Chr11:2,721,948;  Chr11:2,721,951;  Chr11:2,721,957;  Chr11:2,721,962;  Chr11:2,721,964;  Chr11:2,721,974;  Chr11:2,721,978;  Chr11:2,721,982; Chr11:2,721,988 | Imprinting control region |  |
| *SCD1* | F: 5’-GATAAAAGGGGGTTGAGGAAA-3  R: 5’-Biotin-AAACTCTCCCCTCTCTTCT-3  S: 5’-GGGGGTTGAGGAAATA-3 | 4 | Chr10:100,347,893;  Chr10:100,347,899;  Chr10:100,347,907;  Chr10:100,347,934 | +169  +175  +183  +210 | [28] |
| PDX-1 | F: 5’-AGTTGAGAGAGAAAATTGGAATAAAA  GTA-3  R: 5’- Biotin-AATCTCAAAAAAAACCCACAACC  AA-3  S: 5’-AGTGYGGTTAGTTAGGTTAAT-3 | 5 | Chr13:27,920,513;  Chr13:27,920,520;  Chr13:27,920,531;  Chr13:27,920,541; Chr13:27,920,543 | -117  -110  -99  -89  -87 | [12] |
| *FTO* | F: 5’-GAGAGGAGTAYGGGAGAAATATGGT-3  R: 5’-Biotin- CCCTAAACCCCYCCTCCTACATA  TAA-3  S: 5’-TGGGAAATTTTTTTGTGTTAA-3 | 7 | Chr16:53,704,013;  Chr16:53,704,018;  Chr16:53,704,022;  Chr16:53,704,024;  Chr16:53,704,031;  Chr16:53,704,034  Chr16:53,704,036 | +26  +31  +35  +37  +44  +47  +49 |  |
| *PEG3* | F: 5’-ATTTAGGTGTAGAAGTTTGGGTAGT-3  R: 5’-Biotin- ACTCACCTCACCTCAATACT-3  S: 5’-GTTTATTTTGGGTTGGT-3 | 6 | Chr19:56,810,083;  Chr19:56,810,086;  Chr19:56,810,089;  Chr19:56,810,093;  Chr19:56,810,096;  Chr19:56,810,100 | +71  +74  +77  +81  +84  +88 |  |
