# Supplementary material for: DNA methylation of candidate genes in peripheral blood from patients with type 2 diabetes or the metabolic syndrome
Source: PLoS One. 2017 Jul 20;12(7):e0180955. doi: 10.1371/journal.pone.0180955 (PMC5519053; doi:10.1371/journal.pone.0180955)
Supplement: S2 Table — DNA methylation levels of specific CpG loci are shown as median (range). The Kruskal-Wallis Test was performed to test for differences between the three groups. The Wilcoxon Rank-Sum Test was performed to test for differences between two groups of participants. *p≤0.05. (DOCX) [file pone.0180955.s003.docx]

|  | **Median Methylation Level (range)(%)** | | |  | **Pairwise-Test (p-value)** | | | |
| --- | --- | --- | --- | --- | --- | --- | --- | --- |
| **CpG loci** | **Control** | **T2D** | **MetS** |  | **Control vs T2D&MetS** | **Control vs MetS** | **Control vs T2D** | **MetS vs T2D** |
| *PEG3* CpG1 | 27.8  (25.9-64.1) | 27.1  (24.9-46.0) | 28.3  (27.0-64.7) |  | 0.55 | 0.56 | 0.46 | 0.14 |
| *PEG3* CpG2 | 47.7  (45.1-51.5) | 48.8  (39.8-72.8) | 48.9  (47.6-52.1) |  | 0.90 | 0.13 | 0.79 | 0.16 |
| *PEG3* CpG3 | 50.3  (47.3-74.3) | 49.1  (27.4-58.5) | 50.0  (48.9-97.7) |  | 0.57 | 0.49 | 0.33 | 0.07 |
| *PEG3*  CpG 4 | 25.8  (23.9-26.9) | 25.6  (22.6-27.0) | 26.5  (23.0-31.0) |  | 0.81 | 0.56 | 0.56 | 0.34 |
| *PEG3*  CpG 5 | 42.2  (27.3-49.7) | 42.1  (36.9-45.2) | 41.6  (36.7-52.4) |  | 0.89 | 0.91 | 0.96 | 0.79 |
| *PEG3*  CpG 6 | 18.4  (13.1-26.1) | 18.4  (8.8-23.4) | 18.3  (14.3-24.9) |  | 0.65 | 0.86 | 0.95 | 0.88 |
| *KCNJ11*  CpG1 | 4.3  (2.9-5.0) | 4.0  (3.2-5.2) | 3.6  (3.0-5.0) |  | 0.90 | 0.42 | 0.96 | 0.20 |
| *KCNJ11*  CpG2 | 6.9  (6.0-9.9) | 6.8  (5.7-8.6) | 8.2  (6.2-10.2) |  | 0.34 | 0.08 | 0.27 | <0.01* |
| *KCNJ11*  CpG3 | 3.4  (3.2-4.4) | 3.7  (2.3-5.1) | 3.4  (2.7-4.0) |  | 0.54 | 0.59 | 0.66 | 0.45 |
| *PPARγ* CpG1 | 7.0  (5.6-12.5) | 10.5  (6.5-15.0) | 9.2  (5.0-21.3) |  | 0.13 | 0.18 | 0.05* | 0.66 |
| *PPARγ* CpG2 | 11.05  (9.8-13.9) | 11.3  (10.4-16.0) | 13.8  (5.0-21.3) |  | 0.45 | 0.06 | 0.21 | 0.26 |
| *PPARγ* CpG3 | 12.4  (10.1-15.3) | 13.6  (11.3-19.5) | 12.6  (8.2-24.0) |  | 0.46 | 0.34 | 0.16 | 0.06 |
| *PPAR****γ* CpG4** | 9.6  (5.7-12.6) | 10.0  (6.9-16.8) | 10.0  (7.2-19.2) |  | 0.95 | 0.28 | 0.73 | 0.43 |
| *SCD1*  CpG1 | 5.1  (3.8-22.5) | 5.4  (2.9-9.0) | 5.1  (3.1-6.3) |  | 0.82 | 0.62 | 0.44 | 0.23 |
| *SCD1*  CpG2 | 4.1  (2.9-11.6) | 4.5  (2.2-6.5) | 4.7  (3.4-6.0) |  | 0.52 | 0.49 | 0.27 | 0.84 |
| *SCD1*  CpG3 | 5.8  (3.0-16.9) | 6.3  (3.7-8.8) | 6.5  (4.4-6.9) |  | 0.62 | 0.68 | 0.30 | 0.50 |
| *SCD1*  CpG4 | 11.0  (7.9-19.4) | 10.3  (5.7-17.7) | 10.8  (7.3-12.8) |  | 0.99 | 0.97 | 0.60 | 0.69 |
| *KCNQ1*  CpG1 | 39.0  (34.9-41.9) | 38.2  (32.3-43.5) | 37.9  (29.1-42.1) |  | 0.23 | 0.38 | 0.48 | 0.71 |
| *KCNQ1*  CpG2 | 42.8  (39.7-46.4) | 42.9  (35.4-48.5) | 41.4  (40.0-47.1) |  | 0.21 | 0.85 | 0.62 | 0.22 |
| *KCNQ1*  CpG3 | 32.9  (24.8-39.8) | 32.5  (36.0-41.7) | 30.2  (26.4-38.6) |  | 0.60 | 0.24 | 0.69 | 0.45 |
| *KCNQ1*  CpG4 | 42.2  (39.1-45.5) | 42.9  (36.8-50.2) | 42.2  (37.7-47.5) |  | 0.97 | 0.68 | 0.72 | 0.40 |
| *KCNQ1*  CpG5 | 27.7  (22.3-32.0) | 25.7  (22.0-33.7) | 24.3  (22.3-30.4) |  | 0.87 | 0.21 | 0.48 | 0.30 |
| *KCNQ1*  CpG6 | 29.0  (25.2-32.6) | 27.4  (22.2-34.1) | 26.6  (24.9-32.1) |  | 0.63 | 0.18 | 0.33 | 0.47 |
| *KCNQ1*  CpG7 | 39.8  (37.1-42.2) | 39.7  (35.6-46.2) | 38.8  (36.5-40.3) |  | 0.40 | 0.06 | 0.93 | 0.11 |
| *KCNQ1*  CpG8 | 33.4  (31.4-37.1) | 34.4  (27.4-42.2) | 32.6  (29.8-37.1) |  | 0.78 | 0.79 | 0.82 | 0.71 |
| *KCNQ1*  CpG9 | 37.3  (35.0-40.0) | 38.3  (29.1-44.1) | 37.4  (32.9-41.9) |  | 0.69 | 0.79 | 0.35 | 0.31 |
| *FTO*  CpG1 | 5.7  (4.0-7.3) | 5.5  (3.5-8.0) | 6.1  (2.5-12.5) |  | 0.41 | 0.68 | 0.83 | 0.87 |
| *FTO*  CpG2 | 2.5  (1.7-3.4) | 2.8  (1.4-5.1) | 2.9  (1.5-5.6) |  | 0.73 | 0.68 | 0.76 | 0.66 |
| *FTO*  CpG3 | 3.0  (1.2-7.2) | 3.5  (1.0-8.0) | 3.3  (1.6-13.2) |  | 0.15 | 0.40 | 0.34 | 0.86 |
| *FTO*  CpG4 | 1.5  (0.6-2.2) | 1.6  (0.6-4.0) | 1.9  (0.9-4.2) |  | 0.60 | 0.17 | 0.72 | 0.15 |
| *FTO*  CpG5 | 2.3  (1.2-5.3) | 3.1  (1.0-7.7) | 2.8  (1.6-6.3) |  | 0.19 | 0.13 | 0.07 | 0.74 |
| *FTO*  CpG6 | 2.1  (0.9-2.8) | 2.3  (0.9-7.2) | 2.6  (1.2-7.8) |  | 0.32 | 0.01* | 0.11 | 0.21 |
| *FTO*  CpG7 | 1.3  (0.4-3.3) | 1.4  (0.5-4.6) | 0.9  (0.5-4.6) |  | 0.92 | 0.99 | 0.45 | 0.63 |
| *PDX1*  CpG1 | 5.2  (2.2-9.4) | 5.4  (1.4-9.0) | 4.6  (2.7-8.7) |  | 0.40 | 0.52 | 0.42 | 0.87 |
| *PDX1*  CpG2 | 7.7  (4.8-14.3) | 7.8  (2.8-13.3) | 7.2  (5.5-11.9) |  | 0.84 | 0.85 | 0.87 | 0.94 |
| *PDX1*  CpG3 | 3.4  (2.4-5.8) | 3.7  (1.7-6.9) | 3.0  (1.4-6.3) |  | 0.83 | 0.63 | 0.94 | 0.79 |
| *PDX1*  CpG4 | 8.4  (4.1-11.1) | 7.9  (2.5-13.6) | 6.4  (5.0-11.8) |  | 0.65 | 0.62 | 0.73 | 0.62 |
| *PDX1*  CpG5 | 4.4  (1.6-6.9) | 4.2  (2.7-7.1) | 4.4  (3.0-7.1) |  | 0.79 | 0.77 | 0.73 | 0.90 |
| *PDK4*  CpG1 | 56.8  (38.1-80.2) | 50.7  (34.8-81.6) | 53.2  (45.2-64.9) |  | 0.05* | 0.15 | 0.05 | 0.94 |
| *PDK4*  CpG2 | 26.8  (16.7-34.7) | 23.3  (15.4-39.1) | 24.4  (20.2-29.3) |  | 0.53 | 0.65 | 0.54 | 0.98 |
| *PDK4*  CpG3 | 30.8  (22.1-48.3) | 25.0  (19.1-47.4) | 26.7  (21.8-34.3) |  | 0.64 | 0.26 | 0.93 | 0.23 |
| *PDK4*  CpG4 | 27.5  (20.4-37.4) | 23.4  (18.2-63.4) | 25.9  (20.4-32.8) |  | 0.06 | 0.05 | 0.12 | 0.27 |
